# Evaluation of Two Ecosystem Services Provided by a *Pistia stratiotes* Population on the Pacific Coast of South America

**DOI:** 10.3390/biology13080573

**Published:** 2024-07-29

**Authors:** Adela Zamora-Aranda, Héctor Aponte

**Affiliations:** 1 Carrera de Ingeniería Ambiental, Universidad Científica del Sur, Lima 15842, Peru; adelabzamora@gmail.com; 2 Carrera de Biología Marina, Universidad Científica del Sur, Lima 15842, Peru

**Keywords:** carbon storage, coastal wetlands, water lettuce, aquatic plants, forage provision

## Abstract

**Simple Summary:**

Ecosystems store large amounts of carbon, contributing to the control of the gases that lead to climate change. At the same time, certain species provide beneficial materials for the production of fodder or organic fertilizers. This research measured the amount of carbon stored by, and the biomass provision potential of, a water lettuce (*Pistia stratiotes*) population in a disturbed and unprotected Peruvian coastal wetland ecosystem. The results indicated that this population stored 3942.57 tCO_2_ and that a potential 2132.41 tons of biomass could be obtained for fodder. This pioneering research in Peru measured these ecosystem services, demonstrating the potential of this population of floating aquatic plants to provide both services.

**Abstract:**

One of the most fascinating wetlands on Peru’s central coast is the Santa Rosa wetland (Chancay, Lima), an ecosystem threatened by anthropogenic activities. Some of these impacts have led to the uncontrolled growth of *Pistia stratiotes*, an invasive aquatic plant. This study sought to quantify the regulation and provisioning of ecosystem services provided by *P. stratiotes* using carbon storage and the provision of biomass as indicators. To this end, the biomasses of 50 plots measuring 0.0625 m^2^ were weighed and georeferenced and the percentages of dry biomass (%DB) and total organic carbon in the biomass (%C) were quantified. The biomass and its coordinates were entered into ArcGIS and a Kriging interpolation technique was applied to determine the total amount of biomass (B). It was found that *P. stratiotes* stored 3942.57 tCO_2_ and that 2132.41 tons of biomass could be obtained for fodder. The total carbon stored by this aquatic plant represented 28.46% of the total carbon sequestered in the wetland ecosystem by vascular plants, suggesting that its contribution to the carbon cycle is significant. This is the first study to estimate the biomass of a floating aquatic plant population in a coastal Peruvian wetland and is a pioneering study addressing the in situ carbon estimation of Peruvian floating aquatic plants. The results and methods proposed in this research will serve in the evaluation of the potential of ecosystem services among similar populations of floating aquatic species. In addition, the data presented can be used to establish plans for the management and use of this biomass in the production of soil fertilizers and cattle forage.

## 1. Introduction

Ecosystem services are the benefits obtained by people from ecosystems through interactions with nature and are further subdivided into provisioning, regulating, supporting and cultural services [1]. These services are capable of generating food, medicines and energy, contributing to the regulation of all the biogeochemical cycles involved [2]. In this context, the endemic biodiversity of wetlands plays an essential role by offering services with a significant social, economic and environmental value such as tidal control, fiber provision, the maintenance of cultural goods and the cyclical control of nutrients [3]. However, such ecosystems are increasingly being threatened by urban growth, building projects, increases in invasive species and the development of port industries [4]. All of these factors combine to generate the loss and degradation of wetlands, thereby limiting the potential benefits of their ecosystem services among local populations [5]. In Peru, several wetland areas have undergone considerable reductions in area as a result of such threats [6,7,8].

The Santa Rosa wetland (SRW) is one of the most fascinating ecosystems on Peru’s central coast. The diversity of organisms found in this wetland (it has one of the highest concentrations of bird and plant species per unit area on the entire Lima coast) is of major importance [9,10,11]. However, the natural cycles of this wetland have been altered by anthropogenic activities in the area; these include agriculture, solid-waste dumping, land clearance, pig farming and cattle grazing [12]. These activities have resulted in the growth of invasive species, capable of rapidly colonizing and threatening native species, thereby leading to environmental damage [13]. Among the 123 species of vascular plants in the SRW, 2 are introduced species, 31 are invasive and 12 are potential invaders [12]. One of these invasive species is *Pistia stratiotes*.

*P. stratiotes* is a floating aquatic plant species from the Araceae family, which is native to tropical and subtropical regions. Distributed in ponds, freshwater rivers and lakes, *P. stratiotes* forms rosettes up to 30 cm in size composed of thick and fleshy light-green leaves with tiny hairs that protect the plant from dehydration [14]. It has long, thin and fibrous roots and these hang in the water and can branch out and spread in several directions. Its reproduction can be sexual or vegetative through runners or stolons that enable the plant to form large colonies, thereby ensuring rapid propagation [15,16]. Due to the characteristics of its leaves and its capacity for rapid growth, this species is able to adapt to water-stress conditions, giving it an advantage over other aquatic species in terms of survival [17,18,19,20].

In the SRW, *P. stratiotes* is the predominant floating aquatic species [21]. Its uncontrolled proliferation has led to it covering around 80% of the lagoon and it seems likely that excessive nutrient input from drainage and agriculture is responsible for this condition [22]. Alternatives for the control and use of this species have been proposed; however, despite the existing legal framework for its conservation, no large-scale use has been developed to date [23].

It has been demonstrated that aquatic species such as *Eichhornia crassipes*, *Salvinia molesta*, *Azolla* [24,25] and *Elodea potamogeton* [26] have the potential for use as forage, while species such as *Typha domingensis* and *Schoenoplectus americanus* [27], among others, could potentially be used for carbon storage [28]. The carbon capturing of some floating aquatic species in wild conditions is also known; for example, studies of *Eichhornia azurea*, *Nymphaea rudgeana* [29,30], *Pontederia cordata* [31] and *Nymphoides indica* [32] have indicated values of between 3.5 T/ha and 17.5T/ha of carbon. *P. stratiotes* also has the potential to provide ecosystem services such as regulation through carbon storage [33] and provisioning through the production of forage [25]. Clearly, these two services (carbon capture and forage provision) could also be assessed in the population within the SRW. Previous studies have described significant carbon sequestration present in the SRW [27]; however, aquatic populations have not been evaluated, given that the instruments and methodology required are very different from those used for floating aquatic plant populations.

In this context, the aim of this pioneering study—addressing the in situ carbon estimation of Peruvian floating aquatic plants using spatial modeling combined with ecological techniques—was to (a) quantify the biomass of *P. stratiotes* in the SRW, (b) estimate the carbon stored in this habitat and (c) calculate the forage provisioning potential of the biomass.

## 2. Materials and Methods

### 2.1. Study Area

**Figure 1:**
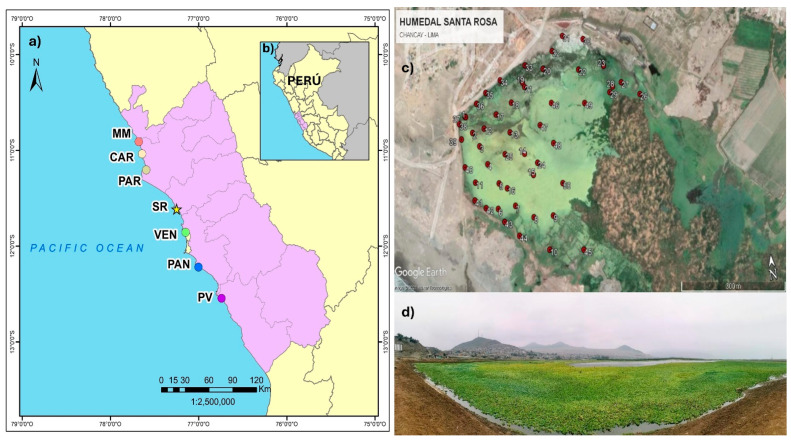
(**a**) Location of Santa Rosa wetland (★) on Peru’s central coast; also shown are other important wetlands, which together constitute an ecological corridor on Lima’s coast. MM: Albufera de Medio Mundo; CAR: Humedal de Carquín; PAR: Laguna El Paraíso; SR: Humedal de Santa Rosa; VEN: Humedales de Ventanilla; PAN: Los Pantanos de Villa; PV: Humedales de Puerto Viejo. In (**b**), the location of SRW in Peru is shown; the purple area is the department of Lima. (**c**) Distribution of plots in the lagoon. Satellite image taken from Google Earth (date of capture: 04 March 2021). (**d**) View of the lagoon, where the population of *Pistia stratiotes* can be seen (date of capture: 24 July 2021).

The study was conducted in the SRW, located in Chancay in the province of Huaral in the department of Lima (Figure 1a,b). This ecosystem receives average annual rainfall of 18 mm, has an average temperature of between 18 and 19 °C and is classified as a subtropical desert [34]. In terms of fauna, 89 species of resident and migratory birds have been registered in the area [10] along with 2 species of reptiles [34]. There are 57 species of vascular plants, of which 98% are herbaceous and 2% are shrubs. Among the aquatic herbs, there are two emergent species and six floating aquatic species [21].

The SRW covers an area of 61 hectares [9], bordered to the north and partially to the northwest by the hill known as Cerro El Cascajo, to the west and partially to the south by the Pacific Ocean, to the south and southeast by agricultural areas (Peralvillo and Salinas Altas) and to the east by Cerro Salinas. Elevated areas are used for grazing. It is fed by water from the Chancay River via a channel and groundwater infiltration [21].

The evaluation was conducted in the main lagoon, which has a surface area of 17.5 hectares and is the primary habitat of *P. stratiotes* in this ecosystem (Figure 1).

### 2.2. In Situ Biomass Assessment

The evaluation was carried out between August and September 2021. A total of 50 plots of 0.0625 m^2^ (0.25 m × 0.25 m) were located at a distance of ≈50 m from each other (Figure 1c). In each plot, the wet biomass of *P. stratiotes* was weighed using a JPSYSTEMS electronic digital hand scale (accurate to 0.01 kg) by removing water from the samples (without mistreating the plants) and excluding any other plant species that were present. Using the wet biomass, a performance curve was used to determine if the number of sampling points was representative of the population. The performance curve stabilized after the 30th sample; however, the measurement was extended to N°50 in order to cover the entire study area (Supplementary Material, Figure S1).

### 2.3. Analysis of Plant Tissue

Six samples of 600 g each of wet biomass were collected and taken to the Universidad Científica del Sur laboratories, where the gravimetric method was applied in order to determine the percentage of dry biomass (%DB) [35].

Four samples of 200 g each were taken to the Soil, Plant, Water and Fertilizer Analysis Laboratory of the La Molina National Agrarian University (LASPAF-UNALM). In order to estimate the percentage of carbon in the biomass (%C), these samples were analyzed using the Walkley and Black method, which measures the organic carbon in samples through oxidation methods, using a sample containing sulfur and chromium [36]. The result was multiplied by a correction factor of 1.32 in order to obtain the oxidizable carbon in the biomass (%CFO) [37].

### 2.4. Statistical Analysis and Spatial Modeling

Once the average wet biomass per plot had been calculated, ARC GIS software (Version 10.8) and Arc Map 10.8 were used to determine the amount of total biomass (B) in the lagoon through interpolation methods. The coordinates and biomass were entered into an information table and a point shapefile was created, after which the limits of the lagoon were established (creating a polygon shapefile) through an image basemap.

The spherical, exponential and circular models were tested using the Kriging interpolation tool (as there were many points with a certain average distance, the ordinary method was chosen) [38,39]. The model with the lowest mean error was selected. The correlation between the measured values and the predictions was also calculated using the selected model. This process has been used in previous studies to calculate plant biomass and carbon stock [40,41]. The modeling values (see the Results section) meant that the ordinary method was used. Using this method, the statistical relationships between the measured points were determined, thereby quantifying the spatial relationship between them. The figure established using the Kriging process was exported to a raster with a pixel size of 0.0625 (representing the plot size). The total biomass of the lagoon (B) was obtained by adding the biomass values of the total pixels.

### 2.5. Carbon Stock and Forage Supply Potential

The carbon stock (CS) was determined by the product of B, %DB and %C using the following equation:

(1) CS=B×%DB×%C

where B is the total biomass (obtained from spatial modeling), %DB is the percentage of dry biomass and %C is the percentage of carbon in the biomass.

Available forage (F) was determined from the dry biomass in the entire lagoon by multiplying B and %DB using the following equation:

(2) F=B×%DB

The amount of protein provided by *P. stratiotes* was obtained by multiplying F and the percent of protein on a dry basis (15.9% [42]).

## 3. Results

Table 1 details the biomass per unit area of each plot. The variability of the biomass in the lagoon was apparent (min = 0 kg; max = 1.4 kg/plot; coeff. var. = 36.90), with a non-homogeneous distribution across the lagoon. The average biomass per plot was 0.82 kg/plot, equal to 13.20 kg/m^2^.

The mean %DB was 5.64% (min = 4.71%, max = 7.32% and coeff. var. = 20.25; for details, see Table 2). The mean %CFO was 38.2% (min = 36.57%, max = 39.63% and coeff. var. = 4.11; details in Table 3). This value showed less variability than the %DB. Using %CFO and the correction factor, the %C was calculated to be 50.42%.

**Figure 2:**
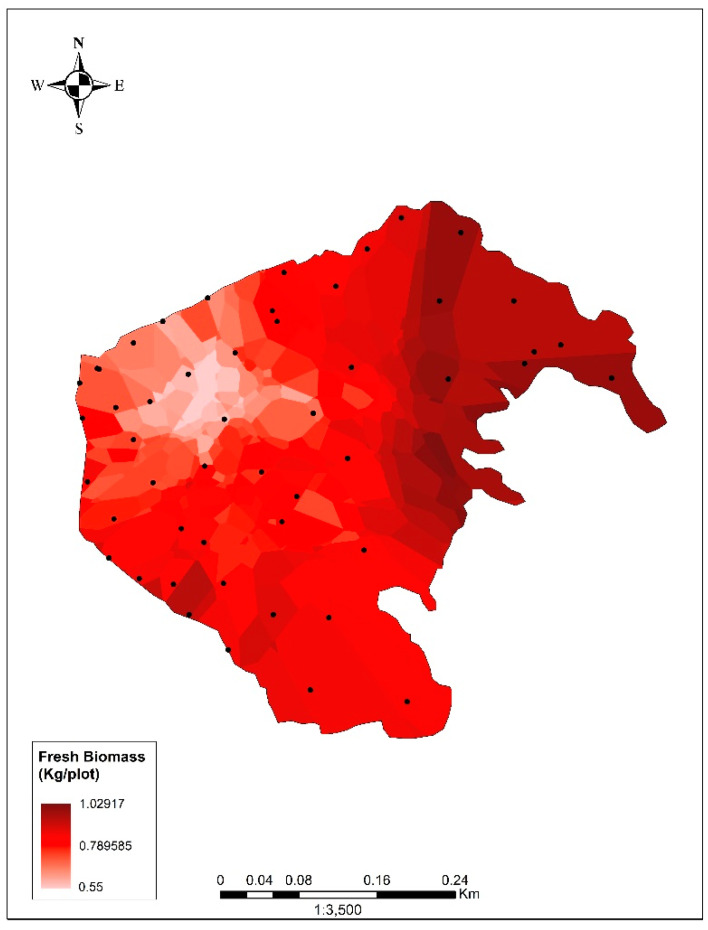
Biomass of *Pistia stratiotes* in the study area. The scale represents the value of fresh biomass (in kilograms) per plot (0.0625 m^2^, corresponding with the area of the pixel used).

B was estimated using the spherical model (error = 0.004; correlation = 0.84; 37,809.99 tons; Figure 2). Other model details are described in Tables S1–S5 and Equation (S1) in the Supplementary Material. Using %DB, %C and B, a total of 1075.25 tons of carbon, equivalent to 3942.57 tons of CO_2_, was calculated. Using %DB and B, forage (F) was found to be 2132.41 tons, equivalent to 339.05 tons of stored protein, resulting in 19.38 Tn/ha.

## 4. Discussion

This study constitutes the first estimation of the biomass and the carbon captured in a floating aquatic plant population of the Peruvian coast. Other similar studies (e.g., [43,44]) did not consider aquatic vegetation, probably because this requires different equipment (for example, a boat and boat oars were required for this sampling). Interestingly, the carbon stored in the area was not homogeneously distributed (Figure 2), which may have been due to the influence of various activities in other lagoon zones. There was water discharge in the northeastern zone from agricultural fields. In the southeastern zone (adjacent to the reed beds and rushes [12,43]), there was the dumping of construction debris and in the northern and western zones, solid waste and wastewater discharges from urban areas were present, along with waste from grazing and other livestock activities [23]. Unlike the eastern and southeastern zones, the western zone was influenced by the harvesting of *P. stratiotes*. This activity was carried out by volunteers, with the aim of reducing the biomass of the species and decongesting the body of water. This could only be carried out on the edges of the lagoon due to the difficulty of access, while the most significant amounts of biomass and stored carbon were concentrated in the central areas of the lagoon, which were the most difficult to access. Due to their nature, the modeling techniques may have had specific limitations; the dynamics of the species in the lagoon were not considered as a variable (these dynamics could have changed the biomass values since the evaluation was conducted). Also, where access was impossible, sections of the lagoon were not evaluated and there was a margin of error in the estimation presented. Nevertheless, the stabilization of the performance curve and the correlation and error coefficients gave us confidence that the estimation was reliable.

The amount of carbon stored by *P. stratiotes* was lower than that stored by other populations of coastal wetland plant species such as *Typha domingensis, Schoenoplectus americanus* and *Sporobolus virginicus*. At the same time, the amount of carbon stored by *P. stratiotes* was higher than the stocks of *Schoenoplectus californicus, Scirpus californicus* and other coastal ecosystems such as Lomas de Amancaes (Supplementary Material, Table S6). Certain communities of tall upright plants store more significant reserves than smaller plants such as *P. stratiotes* (and the mixed vegetation of the SRW); the presence of aerenchyma, the lower percentage of dry biomass and the absence of soil in *P. stratiotes* may explain these differences. The population of *P. stratiotes* in the SRW is one of the largest on Peru’s central coast with no other similar population in the region, thereby making the SRW the ideal laboratory for this study.

A recent study [27] quantified the carbon stored in other plant communities present in the Santa Rosa wetland such as cattails, reeds and mixed meadows (composed of *Cyperus laevigatus* L., *Eleocharis geniculata* (L) Roem. & Schult., *Bacopa monnieri* (L.) Edwall, *Hydrocotyle ranunculoides* L.F., *Paspalum vaginatum* Sw. and *Distichlis spicata* P.M. Peterson & Romasch). When added to the result of this study, the wetland is estimated to store a total of 3778.27 tC, equivalent to 13,828.47 tCO_2_. In this scenario, while the body of water with *P. stratiotes* represents 33% of the total area, it stores 28.46% of the wetland’s total carbon. Therefore, taking into account the economic value per ton of sequestered CO_2_ (USD 6.39 [45]), the value of the carbon stored in the Santa Rosa wetland amounts to USD 87,494.85. If this store was lost, the carbon would return to the atmosphere. In addition, in recent years, satellite images have revealed changes in the biomass over time, together with a eutrophication process [22]. It is, therefore, recommended that measures be adopted in order to protect this and other similar ecosystems on the Peruvian coast and that further measures be implemented to enable the remuneration for ecosystem services like those studied in this research.

The amount of forage calculated demonstrates the potential of this species for the provision of a resource that would contribute to the surrounding livestock communities. However, given its high water content and high percentage of fiber (20.8% [42]), it would constitute a low-quality forage resource. In addition, on a dry basis, *P. stratiotes* has a protein content of 15.9%, which falls below the average value of 18.21% found across other aquatic plant species such as *Lemna minor*, *Wolffia* spp. and *Hydrocharis laevigata* (Supplementary Material, Table S7). Nonetheless, it is essential that the local Cattle Ranchers Association and the Servicio Nacional de Sanidad Agraria (SENASA-Huaral) be involved in order to encourage further research and the harvesting of this plant for cattle ranching purposes as a way of decolonizing the body of water. In addition, *P. stratiotes* can be used for other purposes such as wastewater treatment [46], medicinal and ornamental uses [47], the removal of heavy metals [48] and in fuel production [14,49]. We encourage, therefore, the further evaluation of alternatives for the use of this interesting aquatic species.

## 5. Conclusions

Based on the combined use of field measurements and spatial modeling, we estimated the amount of the *P. stratiotes* biomass in the SRW to be 37,809.99 tons, equivalent to 1075.25 tons of stored carbon, 3942.57 tons of CO_2_, 2134.41 tons of forage and 339.05 tons of stored protein. These measurements constitute a pioneering development in the study of the carbon stock of Peruvian floating aquatic plants. The methodology applied may form the basis for future studies involving similar species and plant biomasses in other aquatic environments. Subsequent studies should consider focusing on the population dynamics of this species in order to assess changes over time in the biomass and the area’s physicochemical conditions. The results of this research may also offer a complementary vision of the role of these species, which are commonly considered to be invasive in nature. These estimates will enable decision-makers to establish plans for the use and management of this plant biomass within the wetland, inspiring new strategies and approaches. The proposal to use plant biomass as forage is just one of the potential uses of this plant and, thanks to this research, it can now be proposed based on more explicit economic values, thereby motivating further support and investment in this area of research.

## Figures and Tables

**Table 1:** UTM coordinates, biomass weight and carbon stored per plot.

N°	X	Y	Weight (kg/plot)	Stored Carbon (kg)
1	0252340	8717547	0.80	0.02
2	0252357	8717508	0.00	0.00
3	0252375	8717475	0.85	0.02
4	0252395	8717431	1.05	0.03
5	0252424	8717384	1.15	0.03
6	0252416	8717327	1.05	0.03
7	0262467	8717328	0.50	0.01
8	0252518	8717296	0.80	0.02
9	0252575	8717293	0.60	0.02
10	0252556	8717219	0.80	0.02
11	0252355	8717394	1.40	0.04
12	0252392	8717514	0.70	0.02
13	0252468	8717496	0.65	0.02
14	0252506	8717442	0.65	0.02
15	0252527	8717391	1.30	0.04
16	0252447	8717370	0.85	0.02
17	0252431	8717542	0.60	0.02
18	0252479	8717564	0.80	0.02
19	0252517	8717607	0.35	0.01
20	0252582	8717632	0.40	0.01
21	0252522	8717596	0.70	0.02
22	0252688	8717617	0.70	0.02
23	0252764	8717617	0.90	0.03
24	0252542	8717417	0.45	0.01
25	0252448	8717448	0.00	0.00
26	0252864	8717538	0.55	0.02
27	0252812	8717572	1.20	0.03
28	0252785	8717565	1.20	0.03
29	0252775	8717553	1.05	0.03
30	0252710	8717687	0.95	0.03
31	0252649	8717702	0.80	0.02
32	0252614	8717670	1.15	0.03
33	0252529	8717646	1.15	0.03
34	0252451	8717620	0.80	0.02
35	0252405	8717596	0.70	0.02
36	0252375	8717574	0.50	0.01
37	0252338	8717548	1.20	0.03
38	0252320	8717533	0.65	0.02
39	0252323	8717497	0.75	0.02
40	0252328	8717432	1.00	0.03
41	0252350	8717354	0.65	0.02
42	0252381	8717333	0.60	0.02
43	0252432	8717296	0.90	0.03
44	0252472	8717260	1.05	0.03
45	0252655	8717207	0.80	0.02
46	0252598	8717549	1.20	0.03
47	0252559	8717502	1.05	0.03
48	0252594	8717456	0.97	0.03
49	0252697	8717537	1.18	0.03
50	0252611	8717362	1.15	0.03

**Table 2:** Percentage of dry biomass (%DB) in the samples.

Sample	%DB
1	6.87
2	5.07
3	4.99
4	7.32
5	4.88
6	4.71
Mean	5.64

**Table 3:** Percentage of readily oxidizable carbon in the biomass (%CFO) of the samples.

Sample	%CFO
1	39.46
2	36.57
3	37.14
4	39.63
Mean	38.2

## Data Availability

The authors confirm that the data supporting the findings of this study are available within the article and its Supplementary Materials.

## Supplementary Materials

The following supporting information can be downloaded at: https://www.mdpi.com/article/10.3390/biology13080573/s1, Table S1. Statistical details of spatial modeling. Table S2. Comparison of the mean error with other Kriging models. Table S3. Spherical model settings. Table S4. Normality test for the model values. Table S5. Correlation between measured values and spherical model predictions. Table S6. Carbon stock in this study and in other Peruvian coastal ecosystems and locations. Table S7. Percentage of protein (P) of different aquatic plants on a dry basis. Values taken from [42], with the exception of the value for *Hydrocharis laevigata*, which was taken from [50]. Figure S1. Performance curve of *Pistia stratiotes* biomass in the SRW. The curve was drawn using the accumulated biomass weight (average biomass of all sampling points) during the establishment of the sampling points. References [27,42,43,44,50,51,52,53,54,55] are cited in the Supplementary Materials.
