# Supplementary material for: Evaluation of Two Ecosystem Services Provided by a Pistia stratiotes Population on the Pacific Coast of South America
Source: Biology (Basel). 2024 Jul 29;13(8):573. doi: 10.3390/biology13080573 (PMC11351440; doi:10.3390/biology13080573)

Evaluation of Two Ecosystem Services Provided by a *Pistia stratiotes* Population on the Pacific Coast of South America

Adela Zamora-Aranda and Héctor Aponte

Supplementary Material

Table S1. Statistical details of spatial modeling.

Measurement	Value
Total sum of points evaluated	44 794 970
Minimum value	0.55
Maximum value	1.03
Sum of points	37 809 996.01
Mean	0.84
Standard deviation	0.09

Table S2. Comparison of the mean error with other Kriging models.

Model	Mean Error
Spherical	0.004
Exponential	0.104
Circular	0.092

Table S3. Spherical model settings.

Model	Spherical
Range	74.8086
Anisotropy	SI
Smaller range	74.8086
Direction	0
Partial threshold	0.0882

Table S4. Normality test for the model values.

Normality Test	Measured Values	Predicted Values
N	50	50
Shapiro–Wilk W-value	0.961	0.9792
p-Value (normal)	0.0975	0.517

Table S5. Correlation between measured values and spherical model predictions.

Correlation Test	Measured Values	Predicted Values
Pearson		0.84
Significance value	0.03	

Table S6. Carbon stock in this study and in other Peruvian coastal ecosystems and locations.

Study Site	Species	Carbon Storage		Reference
		tC/ha	tCO_2/ha	
Humedal Santa Rosa, Chancay, Lima	*Pistia stratiotes*	61.5	225.3	Present study
	Typha domingensis	76.6	280.4	[1]
	Schoenoplectus americanus	63.9	234.1	
	Vega mixta	75.3	275.6	
Refugio de Vida Silvestre Los Pantanos de Villa	*Typha domingensis*	124.7	456.4	[2]
	Cladium jamaicense	161.4	590.7	
	Gramadal, comprising *Distichlis spicata, Sporobolus virginicus* and *Paspalum vaginatum*	85.4	312.6	
	Schoenoplectus americanus	141.0	516.1	
	Phragmites australis	110.6	404.8	
Humedales de Puerto Viejo, Lima	*Schoenoplectus californicus*	28.9	105.8	[3]
	Scirpus americanus	18.6	68.1	
	Paspalum vaginatum	17.0	62.2	
	Salicornia Fruticosa Linneo	6.1	22.3	
Humedales de Ventanilla	*Sporobolus virginicus*	22.8	83.4	[4]
	Scirpus californicus	31.5	115.4	
Other Peruvian Ecosystems				
Lago Titicaca, Puno	*Schoenoplectus tatora*	12.8	46.8	[5]
	Potamogeton strictus	2.9	10.6	
	Myriophyllum elatinoides	0.9	3.3	
	Elodea potamogeton	3.1	11.3	
Lago Chinchaycocha, Junín	*Juncus artivus* var. *andicola*	8.7	31.8	[6]
	Deyeuxia recta Kunth	15.4	56.4	
	Plantago tubulosa	2.7	9.9	
	Eleocharis albibracteata	3.2	11.7	
	Limosella australis	0.6	2.2	

Tillandsial Piedra Campana, Lima	*Tillandsia latifolia*	3.6	13.2	[7]
Lomas de Amancaes	Vegetal biomass	39.3	143.8	[8]

References used in this table:

1. Chavez, R.; Aponte, H. Carbono en el pacífico Sudamericano: reservas en comunidades vegetales de un humedal costero en Perú. *Revista de la Academia Colombiana de Ciencias Exactas, Físicas y Naturales* **2023,** 47(185), 962-976. https://doi.org/10.18257/raccefyn.1925
2. Aponte, H.; Corvacho, MF.; Lertora, G.; Ramírez, D.W. Reserva de carbono en un humedal del desierto costero de Sudamérica. *Gayana Botanica* **2021**, 78(2),184-190. http://dx.doi.org/10.4067/S0717-66432021000200184
3. Palomino Contreras, D.; Cabrera Carranza C. Estimación del servicio ambiental de captura del CO_2 en la flora de los humedales de Puerto Viejo. *Revista del Instituto de Investigación de la Facultad de Minas, Metalurgia y Ciencias geográficas* **2007**, 10(20),49-59. https://revistasinvestigacion.unmsm.edu.pe/index.php/iigeo/article/view/494/419
4. Cieza, M. Estimación de la captura de dióxido de carbono por la flora del Área de Conservación Regional Humedales de Ventanilla. Tesis de pregrado, Universidad Nacional Federico Villarreal, Perú, 2014. https://repositorio.unfv.edu.pe/handle/20.500.13084/716
5. Maldonado-Jiménez, I.; Aparicio-Saavedra, M.E. Estimación del almacenamiento de carbono en la biomasa de macrófitas en la Bahía interior de Puno, lago Titicaca. *Ecosistemas y Recursos Agropecuarios* **2021,** 8(2):e2848. https://doi.org/10.19136/era.a8n2.2848
6. Medrano, Y.; Chupan, M.; Vila, M. Almacenamiento de carbono en especies predominantes de flora en el lago Chinchaycocha. *Apuntes de Ciencia & Sociedad* **2012**, 2(2),110-117. https://doi.org/10.18259/acs.2012013
7. Arévalo, J.; Aponte, H. Almacenamiento de carbono y agua en *Tillandsia latifolia* Meyen en un sector del Tillandsial de Piedra Campana (Lima/Perú). *Ecología aplicada* **2020**, 19(1), 9-15. https://doi.org/10.21704/rea.v19i1.1441
8. Guerrero-Palomino, V.H.; Malca-Rodríguez, D.; Aponte, H. Reservas de carbono en un ecosistema del desierto sudamericano: el caso de Lomas de Amancaes, Lima, Perú. *Revista de la Academia Colombiana de Ciencias Exactas, Físicas y Naturales* **2022**, 46(181),971-984. https://doi.org/10.18257/raccefyn.1760

Table S7. Percentage of protein (P) of different aquatic plants on a dry basis. Values taken from [1], with the exception of the value for *Hydrocharis laevigata*, which was taken from [2].

Species	%P on a Dry Basis
Salvinia auriculata/*S. molesta*	7.9
Chara vulgaris/*Chara* spp.	8.8
Typha latifolia	10.7
Potamogeton spp.	13.1
Eichhornia crassipes	14.8
Alternanthera philoxeroides	15.1
Pistia stratiotes (this study)	15.9
Justicia americana	17.6
Ceratophyllum demersum	17.9
Elodea canadensis	18.0
Sagittaria spp.	18.2
Myriophyllum spp.	20.3
Lemna minor	20.9
Hydrilla verticillata	23.1
Sparganium americanum	23.8
Najas guadalupensis	23.9
Azolla sp.	25.3
Wolffia spp.	27.8
Hydrocharis laevigata	30.0

References used in this table:

1. Tacón, A.G. Proteínas unicelulares. In *Nutrición y alimentación de peces y camarones cultivados*. *Manual de capacitación*. FAO 1989.
2. Ruiz-Merino, M.; Campos-Cuéllar, R.; Germán-Gómez, A.; Aponte, H. (2022). Características, historia natural y aplicaciones de *Hydrocharis laevigata*: Una revisión. *Caldasia* **2022**, 44(2),432–441. https://doi.org/10.15446/caldasia.v44n2.92719

Equation (S1). Spherical model function.

$$F(x) = 0.0406702702711566528X + 0.8171434011785$$

Figure S1. Performance curve of *Pistia stratiotes* biomass in the SRW. The curve was drawn using the accumulated biomass weight (average biomass of all sampling points) during the establishment of the sampling points.

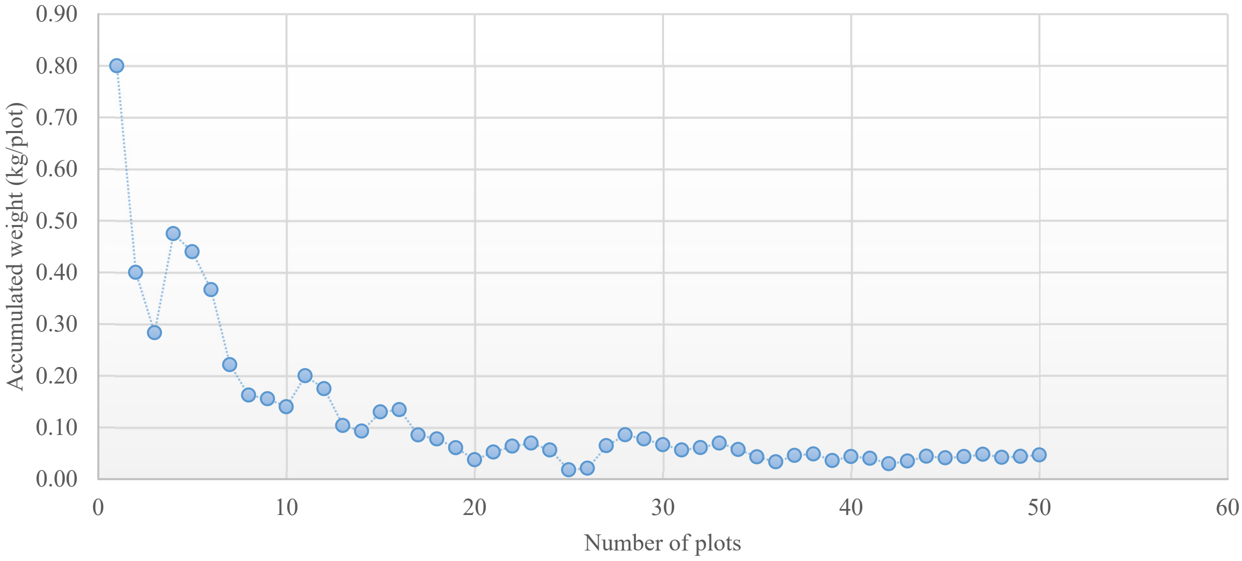

Supplement: Supplementary file 1 [file biology-13-00573-s001.zip › biology-3100074-supplementary.pdf]
